# Antimicrobial Activities of Plant Extracts against *Solanum tuberosum* L. Phytopathogens

**DOI:** 10.3390/molecules27051579

**Published:** 2022-02-27

**Authors:** Aleksandra Steglińska, Anastasiia Bekhter, Paweł Wawrzyniak, Alina Kunicka-Styczyńska, Konrad Jastrząbek, Michał Fidler, Krzysztof Śmigielski, Beata Gutarowska

**Affiliations:** 1 Department of Environmental Biotechnology, Lodz University of Technology, Wólczańska 171/173, 90-530 Łódź, Poland; 217781@edu.p.lodz.pl (M.F.); krzysztof.smigielski@p.lodz.pl (K.Ś.); beata.gutarowska@p.lodz.pl (B.G.); 2 Institute of Natural Products and Cosmetics, Lodz University of Technology, Stefanowskiego 2/22, 90-537 Łódź, Poland; anastasiia.bekhter@dokt.p.lodz.pl (A.B.); konrad.jastrzabek@p.lodz.pl (K.J.); 3 Faculty of Process and Environmental Engineering, Lodz University of Technology, Wólczańska 213, 90-924 Łódź, Poland; pawel.wawrzyniak@p.lodz.pl; 4 Department of Sugar Industry and Food Safety Management, Lodz University of Technology, Wólczańska 171/173, 90-530 Łódź, Poland; alina.kunicka@p.lodz.pl

**Keywords:** plant extracts, natural compounds, garlic extract, clove extract, thyme extract, subcritical carbon dioxide extraction, biopesticide, potato seeds, plant pathogens

## Abstract

The purpose of the study was to select an environmentally friendly plant biopesticide to protect seed potatoes against phytopathogens. The scope included the evaluation of the antimicrobial activities of 22 plant water extracts, 22 water-glycol extracts, and 3 subcritical carbon dioxide extracts using the agar diffusion method against 10 potato phytopathogens. For the most effective extracts, minimal inhibitory concentration (MIC), chemical composition analysis by gas chromatography–mass spectrometry and in situ assays on seed potatoes were performed. Garlic water extract was finally selected as the most effective in phytopathogen growth inhibition, both in vitro and in situ, with MIC values ranging between 6.3–25 mg/mL. 5-Hydroxymethylfurfural was determined to be the main component of this extract (33.24%). Garlic water extract was proposed as a potential biopesticide against potato phytopathogens.

## 1. Introduction

Potato (*Solanum tuberosum* L.) is the third-most consumed food plant in the world, following wheat and rice. World potato production is growing constantly [1]. Poland is the seventh-largest producer in the world and the second-largest in Europe. The harvest in 2020 was estimated at 9 million tons, which is 40% more than the harvest in 2019, while the cultivation area was about 300,000 ha [2]. Crop pathogens (phytopathogens) reduce the quality and yield of agricultural production. They are responsible for huge economic losses and reduce food security nationally and globally. In the case of potatoes, yield losses are estimated at 17.2% worldwide [3]. Unfavourable climatic changes (heat and droughts) increase the sensitivity of crops to phytopathogen infestations [1].

The most dangerous bacterial phytopathogens of potato tubers are *Clavibacter michiganensis* subsp. *Michiganensis,* causing bacterial ring rot disease [4]; *Pectobacterium carotovorum*, the cause of soft rot [5]: *Ralstonia solanacearum,* leading to potato wilting: and *Streptomyces scabiei,* inducing the potato common scab [6]. In turn, the main fungal phytopathogens are *Fusarium* spp., associated with Fusarium dry rot; *Rhizoctonia solani*; *Colletotrichum coccodes*; and the *Alternaria* and *Phoma exigua* moulds, which develop during potato storage [7,8,9,10].

The basis of healthy seed potato production is prevention by the elimination of infection sources, introducing crops of increased resistance and, essentially, the appropriate seed potato storage from harvest to plant. Seed potatoes should be stored in optimal temperature (3–4 °C) and humidity (90–95%). These storage conditions prevent potato mass decline, chemical composition change, loss after germination, and, importantly, microorganism infestation [11] during several months of storage. Although synthetic fungicides effective against phytopathogens exist, their excessive usage leads to the development of phytopathogen resistance to chemicals and long-term negative effects on human health [12,13]. Nowadays, the global trend seems to be a shift towards reducing the use of synthetic fungicides, and thus there is a strong and growing tendency to seek safer and more ecological alternatives to combat plant diseases.

An environmentally friendly solution may be the use of plant extracts. These are composed of the plant cells’ secondary metabolites. Although the quality and quantity of these compounds depend on plant species, environmental growth conditions, pathogen incidence, harvesting season, and method of extraction, their biopesticidal characteristics are usually broad and targeted against a variety of plant pathogens. Moreover, they are biodegradable and do not cause severe harm to the environment. Therefore, plant extracts can serve as a natural alternative to synthetic fungicides for potato treatment against post-harvest storage phytopathogens [13,14].

In order to obtain plant extracts containing the desired bioactive compounds, it is important to select the appropriate extraction method. The common solvents used for extraction, such as hexane, methanol, acetone, and chloroform, are carcinogenic; therefore, they should not be used in products for crop protection. This research focused mainly on water and glycol-water extraction, which remain safe to human health [15]. The addition of the sonification process allows the receipt of a higher yield of extraction because of plant cell-wall destruction. The most common method of obtaining essential oils is steam distillation although it causes the decomposition of compounds that are sensitive to temperature [14]. An alternative may be a supercritical carbon dioxide extraction, a novel—and recognised as green—technology of plant extraction. Avoidance of thermal degradation, as well as solvent contamination, is the main advantage of this method [15]. Several studies reported the successful usage of this technique to prepare extracts from *Thymus vulgaris* [16], *Satureja montana* [17], and *Borago officinalis* L. [18].

Plant extracts that are reported to exhibit antimicrobial activity against potato phytopathogens from genus *Fusarium* include *Caryophyllus aromaticus* L. (clove), *Lavandula angustifolia* L. (lavender), *Mentha piperita* L. (peppermint), *Rosmarinus officinalis* L. (rosemary) and *Thymus vulgaris* L. (thyme) extracts [19]. *Rhizoctonia solani* development inhibition was confirmed in several studies for *Nigella sativa* L. (blackseed) [20], *Allium sativum* L. (garlic) [21], *Urtica dioica* L. (nettle) [9], *Allium cepa* L. (onion) [22], and *Salvia officinalis* L. (sage) [23]. *Alternaria* species growth was inhibited as shown in previous studies by *Carum carvi* L. (caraway) [24], *Allium sativum* L. [25], *Thymus vulgaris* L. [26], and *Curcuma longa* L. (turmeric) [25]. *Carum carvi* L. has been additionally mentioned as a *Pectobacterium carotovorum* growth inhibition agent [27].

In the study, we performed an extensive screening of plant extracts with potential for application as biopesticides for potato protection against phytopathogens, aiming to select the optimal extraction method in terms of antimicrobial properties and economic efficiency. Moreover, the compounds of the extracts that most effectively inhibited the growth of phytopathogens were identified, and application tests were performed on seed potatoes. The operating range included the evaluation of the antimicrobial properties of 22 water plant extracts, 22 water-glycol plant extracts, and 3 plant extracts obtained by subcritical carbon dioxide extraction (SCDE) against 10 seed-potato phytopathogens. The most effective plant extracts were chosen: 2 water and 2 SCDE extracts and their chemical characteristics and MIC values. In situ tests of the selected extracts were performed on seed potatoes.

## 2. Results and Discussion

### 2.1. In Vitro Evaluation of Antimicrobial Activities of Plant Extracts against Potato Phytopathogens

The antimicrobial activity of 22 water plant extracts (WE), 22 water-glycol plant extracts (WGE) and the subcritical CO_2_ extracts (SCDE) of 3 plants, namely blackseed, thyme, and caraway, were tested against 10 potato phytopathogens.

**Figure 1:**
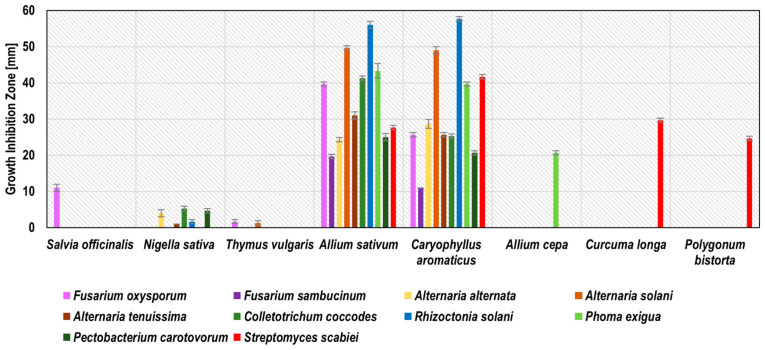
Activity of plant water extracts against potato seed phytopathogens measured as growth inhibition zones in the agar-well diffusion method. The graph only includes plants that showed inhibition of at least one phytopathogen.

The susceptibility to phytopathogens varied greatly and was influenced by the pathogen–extract relationship. It was shown that eight out of all tested water extracts inhibited the growth of at least one strain of potato phytopathogen: sage, blackseed, thyme, garlic, clove, onion, turmeric, and bistort (Figure 1). The zones of phytopathogen growth inhibition ranged from 1.0 ± 0.0 mm (blackseed WE extract against *A. tenuissima*) up to 57.6 ± 0.6 mm (clove WE against *R. solani*). Garlic and clove WE have demonstrated the broadest spectrum of antimicrobial activity, inhibiting the growth of all 10 tested phytopathogens.

The antibacterial property of garlic extracts against several human pathogens, including Pseudomonas aeruginosa, Staphylococcus aureus, Escherichia coli, Salmonella spp., and Streptococcus mutans has already been described [28,29,30,31,32]. Although the activity of garlic extracts against potato phytopathogens is under-reported, a garlic WE was active against *Alternaria solani* [33], while chloroform, hexane, methanol [34], and water [22] extracts inhibited the growth of *Rhizoctonia solani*. In our work, garlic WE was active against *F. oxysporum* (19.7 ± 0.6 mm) and against *R. solani* (56.0 ± 1.0). To the best of our knowledge, there are no literature data on clove WE activity against potato phytopathogens. Several studies, however, have reported the antifungal activities of clove essential oil and ethanol-water extracts against potato phytopathogens. Clove essential oil exhibited antifungal activity against *F. oxysporum* [19,21,35,36] and *Alternaria alternata* [37], while ethanol-water extract inhibited the growth of *Rhizoctonia solani* [20]. In our study, the phytopathogen growth inhibition zones caused by clove WE ranged from 11.0 ± 0.0 mm against *F. sambucinum* to 57.6 ± 0.6 mm against *R. solani.*

The most susceptible phytopathogens to garlic and clove WE were the moulds *R. solani* and *A. solani*, while the weakest activities of these extracts were demonstrated against *F. sambucinum* and *P. carotovorum*. Based on Ponce et al. [38], garlic and clove extracts were considered to be active (inhibition zone diameter 9–15 mm; clove WE against *F. sambucinum*) or very active (inhibition zone diameter >15 mm; the other combinations of extract and phytopathogen). Onion WE against *P. exigua* (20.1 ± 0.6 mm) as well as turmeric and bistort WE against *S. scabiei* (29.7 ± 0.6 and 24.6 ± 0.6 mm, respectively) were recognized as very active. According to several studies, the growth of *R. solani* might be inhibited by turmeric [25], nettle [9], sage [9,22,23], and onion water extracts [22]; however, our study indicates no activity of these extracts against *R. solani*.

On the contrary, our observations confirmed Abd-El-Khair et al. [22], who point out that peppermint WE was inactive against *R. solani*. An earlier study also reported the antifungal activity of blackseed ethanol-water extract against *R. solani* [20], probably due to the addition of ethanol as a solvent, enriching the extract in active compounds. Discrepancies in antifungal activity may result from differences in the chemical composition of individual extracts depending on plant variety, storage methods, or the exact extraction procedure. The detailed results of phytopathogen growth inhibition by water extracts are presented in Figure 1 and Table S1.

**Figure 2:**
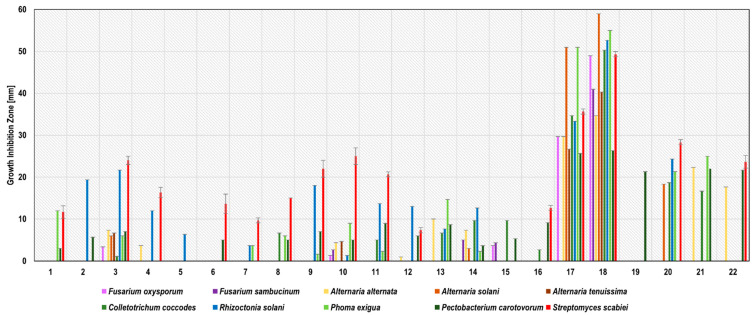
Activity of plant glycol-water extracts against potato seed phytopathogens measured as growth inhibition zones in the agar-well diffusion method. 1—common yarrow; 2—peppermint; 3—sage; 4—common horsetail; 5—nettle; 6—dandelion; 7—couch grass; 8—perforate St. John’s wort; 9—rosemary; 10—common hop; 11—summer savoury; 12—caraway; 13—blackseed; 14—thyme; 15—lavender; 16—horseradish; 17—garlic; 18—clove; 19—onion; 20—turmeric; 21—bistort; and 22—common knotgrass.

Glycol-water plant extracts expressed a wider range of activity compared to the water extracts of the same plant. All of the 22 glycol-water extracts revealed antimicrobial activity against at least one strain of the phytopathogens tested (Figure 2). The zones of growth inhibition ranged from 1.0 ± 0.0 mm (caraway WGE against *A. alternata*) to 59.0 ± 1.0 mm (clove WGE against *A. solani*). As in the case of water extracts, the most active glycol-water extracts were garlic and clove, which inhibited the growth of 9 (no activity against *F. sambucinum*) and 10 phytopathogens, respectively. The zones of growth inhibition estimated for garlic WGE ranged from 25.7 ± 0.6 against *P. carotovorum* to 51.0 ± 1.0 mm against *P. exigua* and *A. solani*. For clove WGE, these values ranged from 26.3 ± 0.6 mm against *P. carotovorum* to 59.0 ± 1.0 mm against *A. solani*. The most sensitive phytopathogens to these two glycol-water extracts were *A. solani*, *P. exigua*, *R. solani,* and *C. coccodes*, while the least sensitive were *P. carotovorum* and *A. alternata*. The following WGE were classified as very active: turmeric against *A. solani*, *C. coccodes*, *R. solani*, *P. exigua,* and *S. scabiei*; bistort against *A. alternata*, *C. coccodes*, *P. exigua,* and *P. carotovorum*; common knotgrass against *A. alternata*, *P. carotovorum,* and *S. scabiei*; sage and rosemary against *R. solani* and *S. scabiei*; peppermint against *R. solani*; horsetail, hop, and summer savoury against *S. scabiei*; onion against *P. carotovorum*. No studies were found regarding glycol-water extracts against potato phytopathogens. This article is the first such extensive research in this field. Previous studies reported inhibition of *A. alternata* growth by peppermint and lavender methanol-water extract [10] and peppermint essential oil [26]; however, due to a different type of extract, these findings were not reflected in the present work. The detailed results of phytopathogen growth inhibition by water-glycol extracts are presented in Figure 2 and Table S2.

**Figure 3:**
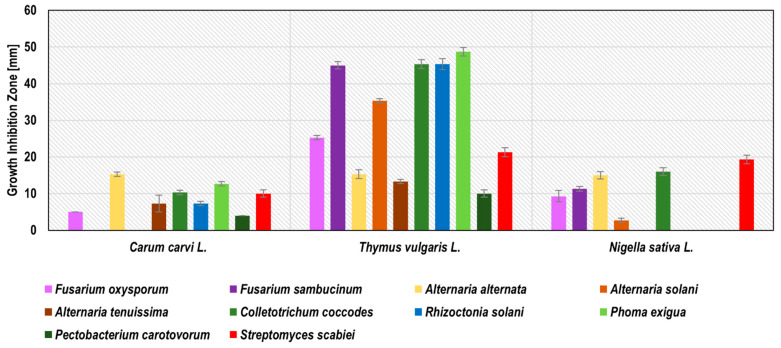
Activity of plant subcritical carbon dioxide extracts (SCDE) extracts against potato seed phytopathogens measured as growth inhibition zones in the agar-disc diffusion method.

Blackseed, thyme, and caraway subcritical carbon dioxide extracts (SCDE) were tested against potato phytopathogens. Only these three plants were successfully used for subcritical carbon dioxide extraction, probably due to the high level of oil fraction in the seeds of these plants. All of these SCDEs inhibited the growth of at least six phytopathogens (Figure 3). The growth inhibition zones ranged from 3.1 ± 0.6 mm for the blackseed SCDE against *A. solani* to 48.4 ± 1.7 mm for the thyme SCDE against *P. exigua*. Thyme SCDE showed the broadest spectrum of antimicrobial activity by inhibiting the growth of all 10 phytopathogens tested. The most susceptible to thyme SCDE were *P. exigua* (48.4 ± 1.7 mm), *R. solani*, *C. coccodes,* and *F. sambucinum* (45.2 ± 1.5, 45.2 ± 1.5, and 45.0 ± 0.6 mm, respectively), while the least susceptible were *P. carotovorum* and *A. tenuissima* (10.0 ± 1.0 and 13.2 ± 0.6 mm, respectively). Our research confirmed the antifungal activity of thyme SCDE against *A. alternata,* as previously described by Perina et al. [39], Soković et al. [26], and Puškárová et al. [37] for thyme essential oil/mL. Zambonelli et al. [40] indicated the antifungal activity of thyme essential oil against *R. solani*, which coincides with our results for thyme SCDE. Diánez et al. [19] present the antifungal activity of thyme essential oil against *F. oxysporum*, which is compatible with our research for thyme SCDE. Caraway SCDE inhibited the growth of eight phytopathogens. The best effect was observed against *A. alternata* (growth inhibition zone 15.3 ± 0.6 mm) and *P. exigua* (growth inhibition zone 12.6 ± 0.6 mm). Convergent results were obtained in previous studies for caraway essential oil, for which antimicrobial activity has been demonstrated against *P. carotovorum* [27] and *A. alternata* [24]. Blackseed SCDE inhibited the growth of six phytopathogens, with the largest inhibition zones against *S. scabiei* (19.2 ± 1.2 mm) and *C. coccodes* (16.1 ± 1.2 mm). Based on Ponce et al. [28], thyme SCDE was considered as active against eight tested pathogens (*F. oxysporum*, *F. sambucinum*, *A. alternaria*, *A. solani*, *C. coccodes*, *R. solani*, *P. exigua,* and *S. scabiei*); caraway SDCE was recognised as very active against *A. alternata,* and blackseed SCDE against *C. coccodes* and *S. scabiei*. The detailed results of phytopathogen growth inhibitions by SCDE are presented in Figure 3 and Table S3.

Glycol-water extracts exhibited in general a wider spectrum of activity against the potato phytopathogens than water extracts. The exceptions were garlic and clove extracts, which inhibited a similar number of pathogens regardless of the solvent used. The greatest differences in antimicrobial activity were observed for SCDE thyme extracts compared to both WE and WGE. Thyme SCDE was recognized as active or very active against all 10 tested phytopathogens, while both WGE and WE only against 2 pathogens. These differences were similar for caraway and blackseed SCDE (active or very active against 4 and 5, respectively) in comparison to WE (not active or very active), but smaller for WGE (active against 1 and 3 pathogens, respectively).

The Minimal Inhibitory Concentration (MIC) and Minimal Bactericidal/Fungicidal Concentration (MBC/MFC) were determined for garlic and clove water extracts and for thyme and caraway subcritical CO_2_ extracts that were the most effective against the phytopathogens. Further experiments on glycol-water extracts were abandoned due to their negative impact on the morphological features of seed potatoes (data not published). The detailed results are presented in Table 1.

MIC values of garlic WE vary between 6.3 and 25 mg/mL, and the most sensitive were the moulds *A. solani* and *P. exigua* (MIC 6.3 mg/mL), followed by *F. oxysporum*, *F. sambucinum*, *C. coccodes*, *R. solani* and Gram-positive bacteria *S. scabiei* (MIC 12.5 mg/mL). The growth inhibition of two moulds of the genus *Alternaria*: *A. alternata* and *A. tenuissima,* as well as bacteria *P. carotovorum,* was possible at a garlic WE concentration not lower than 25 mg/mL. The lower MIC value of garlic WE compared to Gram-negative bacteria *P. carotovorum* was noted against other representatives of the *Enterobacteriaceae* family, *E. coli* and *Salmonella typhi,* by Iwalokun et al. [41] (MIC 20.8 and 21.8 mg/mL, respectively). In the same study, MIC values for *S. aureus* and *Streptococcus pneumoniae* were 27.1 and 30.3 mg/mL, respectively, which is more than two times higher than those obtained in our work for *S. scabiei*, another representative of Gram-positive bacteria. In the present work, MBC/MFC values of garlic WE ranged from 12.5 to 25 mg/mL against the tested microorganisms, with the exception of *A. tenuissima,* for which no MFC was detected.

MIC values of clove WE were found to be 50 to 75% lower than those of garlic WE and vary between 3.1 and 6.3 mg/mL for all the phytopathogens tested. On the contrary, the MBC/MFC values were more diversified. The lowest were recorded against *A. solani*, *R. solani,* and *P. exigua* (6.3 mg/mL), and the highest against *P. carotovorum* (25 mg/mL). As mentioned above, there are no previously reported data on clove WE activity against potato phytopathogens. There are, however, some reports on clove essential oil action against selected fungi. Puškárová et al. [37] have not estimated the MFC value (MIC was equal to 0.025%) of clove essential oil against *A. alternata*, which is similar to our results on MFC determination obtained for clove WE (in our work, MIC was 252 times higher). Rana et al. [36] determined the MIC of clove essential oil against *F. oxysporum* as equal to 10 µL/mL, about 630 times lower than that observed in our work for clove WE. In another study, Sharma et al. [21] reported MIC 31.25 ppm and MFC 125 ppm of clove essential oil against *F. oxysporum*, 2016 and 1000 times lower than the values obtained in the present work for clove WE, respectively. The weaker antifungal action of clove WE compared to clove essential oil may be attributed to the higher concentration of antifungal components in pure essential oil than in water extract.

MIC values of thyme SCDE ranged between 2.9 and 11.5 mg/mL. The lowest MIC values were recorded against *C. coccodes* and *S. scabiei* (2.9 mg/mL), while the highest were against *A. alternata*, *A. tenuissima,* and *P. exigua* (11.5 mg/mL). In the earlier studies of Puškárová et al. [37] and Soković et al. [26], however, thyme essential oil revealed antifungal activity at a much lower concentration (MIC 0.025% and MIC 0.25 μL/mL, respectively). It can be assumed that this is related to the differences in the qualitative and quantitative composition of the essential oil and the subcritical carbon dioxide extract.

The MIC values for caraway SCDE were the highest of all the extracts tested. The growth of five phytopathogens (*A. alternata*, *A. tenuissima*, *P. exigua*, *P. carotovorum,* and *S. scabiei*) was inhibited by only 0.9 mg/mL undiluted extract. The lowest value was observed against *F. oxysporum,* and the minimum inhibitory concentration was 22.5 mg/mL. The MIC values of caraway SCDE against *P. carotovorum* and *A. alternata* recorded in this work were significantly higher than the values of caraway essential oil obtained in the work of Iacobellis et al. [27] (910 µg) and Begum et al. [24] (50 ppm).

Due to the high activity and broad spectrum of action, in terms of future research on seed potatoes, we recommend the use of water extracts from garlic and clove, as well as caraway and thyme subcritical carbon dioxide extracts. The use of other extracts that exhibited high antimicrobial activity but not against such a wide phytopathogen spectrum is also worth considering, e.g., onion WE (antifungal activity against *P. exigua*), turmeric and bistort WE (against *S. scabiei*), and blackseed SCDE (against *C. coccodes* and *S. scabiei*).

### 2.2. Chemical Composition of Selected Extracts

The chemical composition of garlic and clove water extracts, as well as that of thyme and caraway subcritical carbon dioxide extracts, was identified. The results are presented in Table 2, Table 3, Table 4 and Table 5.

A total of 33 (97.54%) compounds of clove water extract were identified and eugenol (allylbenzene class of chemical compounds) was determined to be the main component (82.39%). This result is in accordance with the results reported for different types of clove extracts by many authors. In other studies, eugenol constituted 53.9% [42], 75.41% [21], or even 85% [35] of clove oil and 52.88% of subcritical CO_2_ extract [43] and was the principal compound in all of them. The eugenol content indicated in the literature was a result of both the origin of the plant material and the fact that the analytical method used (GC-MS) gives the relative content of the ingredients, and not the actual one. This is confirmed by the lower content of eugenol in carbon dioxide extract (52,88%) because this extraction method shows the full organic profile of the plant matrix and consequently a greater number of compounds. The relative content of eugenol was therefore lower in carbon dioxide extract than in essential oils of clove. The presented investigation also revealed the presence of eugenol acetate (4.56%) and caryophyllene (2.49%). These compounds are also reported in the above-mentioned studies of clove extracts in different concentrations. It is well documented that the antimicrobial activity of clove extracts is mostly related to high eugenol content, and the mode of action is based on cytoplasmic membrane disruption, inhibition of some microbial enzymes, and the negative effect of ions and ATP transport [44].

A total of 42 (83.01%) components of garlic water extract were identified, and 5-hydroxymethylfurfural (33.24%) was observed in the highest quantity. This compound was previously found in black garlic subjected to heat treatment [45] and in three Chinese varieties of fresh garlic (4.88, 26.78 and 47.10%); however, no antimicrobial activity has yet been reported for 5-hydroxymethylfurfural. No allicin was observed in our garlic WE, probably due to its instability and quick transformation into other sulphur components [46]. Small quantities of diallyl disulphide (1.58%), dimethyl trisulphide (0.95%), 3-vinyl-1,2-dithiacyclohex-4-ene (0.44%), allyl methyl trisulphide (0.41%), and others were determined and might be jointly responsible for the strong antimicrobial activity of garlic WE against the tested potato phytopathogens [13].

In total, 63 (95.26%) of the components of thyme subcritical CO_2_ extract were identified and thymol (monoterpenoid phenol derivative of *p*-cymene; 48.54%) was determined to be dominant. This result is confirmed by numerous studies, which verify the presence of thymol in thyme essential oils at a level of 39.14 [47], 47.9 [48], and 48.9% [26]. Some studies [49] reported another monoterpenoid phenol, thymol isomer carvacrol, as a major component of thyme essential oil, which in the current study was present at a level of 3.16%. Also, the presence of *p*-cymene (8.53%) was reported in the above-mentioned studies. The antimicrobial activity of thymol and carvacrol is revealed in the work of Perina et al. [39] and Sim et al. [50]. Their mode of antimicrobial action included the disruption of cell walls, leading to leakage of cell components and, consequently, to lysis. Other reported mechanisms include disturbances in protein synthesis and quorum sensing [51].

Based on 33 (99.19%) of the compounds identified in caraway subcritical CO_2_ extract, (+)-carvone (terpenoid; 52,14%) and d-limonene (cyclic monoterpene; 37.17%) were determined as the two main components. These results are similar to those obtained by Iacobellis et al. [27], where carvone (23.3%) and limonene (18.2%) were detected as the principal components of caraway essential oil; however, other compounds found in high quantities in this work, such as germacrene and trans-dihydrocarvone, were found only in traces in the caraway SCDE in the present study. Also, Argañosa et al. [52] and Putievsky et al. [53] indicated carvone and limonene as the dominant components in caraway essential oil. A different composition, however, was reported by Begum et al. [24], where thymol (48.20%), o-cymene (19.29%), and γ-terpinen (17.61%) were the main components.

### 2.3. In Situ Evaluation of Antimicrobial Activities of Selected Plant Extracts against Potato Phytopathogens

**Figure 4:**
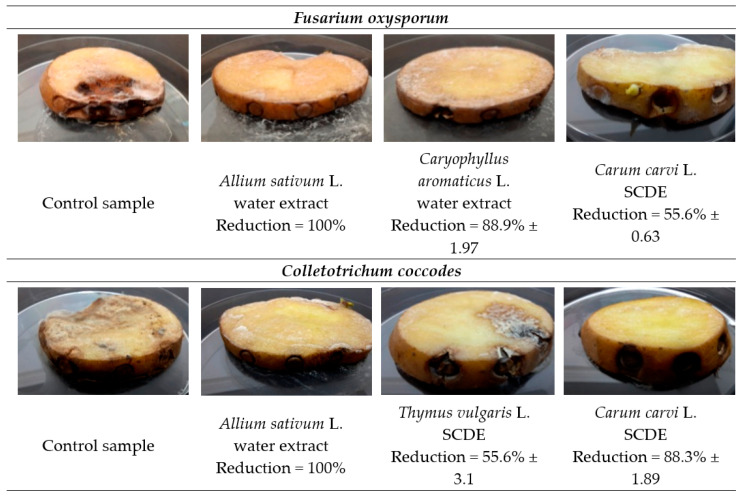
Inhibition of *F. oxysporum* and *C. coccodes* infestation of potatoes cv. Impresja treated with plant extracts.

The in situ antimicrobial activities of garlic and clove WE, as well as those of caraway and thyme SCDE, on seed potatoes were evaluated. The results are presented in Table 6 and Figure 4.

The in situ assay revealed that garlic WE reduced potato infestation of *F. oxysporum*, *F. sambucinum*, *A. solani*, *C. coccodes*, *R. solani*, *P. exigua,* and *S. scabiei*. It was, however, ineffective against *A. alternata*, *A. solani,* and *P. carotovorum*, while the in vitro assay performed using the agar-well diffusion method indicated significant growth inhibition. Nashwa et al. [33] reported lower reduction of *A. solani* growth on tomatoes (61.7 and 71.7% reduction), but the WE concentrations of garlic used were also lower (1 and 5%, respectively). In our work, a 100% reduction of *A. solani* infestation was achieved by non-diluted garlic WE.

Clove WE treatment inhibited *F. oxysporum* (88.9%) and *A. tenuissima* (100%) infestation of seed potatoes satisfactorily in the in situ assay. This treatment, however, increased the spread of *A. solani* (+25%), *C. coccodes* (+10%), *R. solani* (+93.8%), *P. exigua* (+95%) and *S. scabiei* (+75%), even though the in vitro assessment of clove WE by the agar-well diffusion method demonstrated positive results of pathogen growth inhibition. Similar findings were reported by Muñoz Castellanos et al. [35] for clove essential oil, where the oil at a lower concentration (350 ppm) reduced *F. oxysporum* growth on tomatoes more effectively than at 450 ppm. Opposite results are reported for CO_2_ clove extract by Šernaitė et al. [43], where the clove SCDE, at a concentration of 6 mL/L, increased the infection of grey mould on strawberry leaves, while at a twice-higher concentration significantly reduced it.

The antimicrobial activity of thyme and caraway SCDEs at concentrations equal to exact MIC values was tested against three seed potato phytopathogens. The extracts demonstrated reduction of *F. oxysporum* (33.3 and 55.6%, respectively) and *C. coccodes* (55.6 and 83.3%, respectively) infestation on seed potatoes but were totally ineffective against *P. carotovorum* bacteria (0%). To the best of our knowledge, the antimicrobial effects of thyme and caraway extracts against seed potato phytopathogens have not yet been studied in in situ assays. Nikkhah et al. [47], however, reported on the antifungal activity of thyme essential oil in a concentration equal to 625 µg/mL against *Botrytis cinerea*, which causes spoilage on pear fruit.

## 3. Materials and Methods

### 3.1. Plant Material and Extracts Preparation

Plant material from 22 different plants, presented in Table 7, was purchased from Dary Natury Sp. z o. o., Grodzisk, Poland and Zakład Zielarski KAWON–HURT Nowak sp.j., Gostyń, Poland.

#### 3.1.1. Water Extracts (WE)

Twenty grams of finely ground plant material was poured into 200 mL of water at 100 °C and left covered without stirring for 1 h. The obtained extracts were then sonicated (40 kHz, 25 °C) for 30 min and filtered under reduced pressure.

#### 3.1.2. Water-Glycol Extracts (WGE)

Eight grams of ground plant material was poured into 200 mL of extractant 1:4 water: propylene glycol (*v*/*v*) (Chempur, Piekary Śląskie, Poland) and stirred for 3 h at room temperature. The obtained extracts were then sonicated (40 kHz, 25 °C) for 30 min and filtered under reduced pressure.

#### 3.1.3. Subcritical Carbon Dioxide Extracts (SCDE)

The extraction was performed in an apparatus designed and constructed at the Faculty of Process and Environmental Engineering at the Lodz University of Technology, Poland. Between 64.5 and 101.4 g of finely ground seed powder was poured into a 280 cc stainless steel pressure extraction vessel. The system was operated at a constant pressure of 31.4 ± 0.02 MPA, with the temperature maintained at 28 ± 1 °C. A liquid CO_2_ pump provided a constant carbon dioxide flow at a rate of 7 cc/min, while an automatic back pressure regulator controlled pressure within the extraction vessel. The extract was collected in a glass trap after reduction of CO_2_ pressure to 0.1 MPa. The whole extraction process lasted for ~6 h, which was sufficient for complete extraction. In all experiments, the ratio of CO_2_ used for extraction to the weight of the raw material was between 25 and 30.

### 3.2. Potato Phytopathogens

The fungal species *Colletotrichum coccodes* DSM 62126, *Phoma exigua* DSM 62040, *Fusarium sambucinum* DSM 62397, *Rhizoctonia solani* DSM 22843, *Alternaria tenuissima* DSM 63,360, and the bacterial species *Streptomyces scabiei* DSM 40,778 were obtained from the German Collection of Microorganisms and Cell Cultures GmbH (DSMZ, Braunschweig, Germany). *Pectobacterium carotovorum* PCM 2056 was purchased from the Polish Collection of Microorganisms of the Hirszfeld Institute of Immunology and Experimental Therapy of the Polish Academy of Sciences (Wrocław, Poland). *Alternaria alternata* ŁOCK 408 was acquired from the Collection of Pure Cultures of Industrial Microorganisms ŁOCK at the Lodz University of Technology (Łódź, Poland). *Alternaria solani* Z184 and *Fusarium oxysporum* Z154 isolates were kindly provided by Prof. Jadwiga Śliwka from the Plant Breeding and Acclimatization Institute (IHAR)—National Research Institute (Radzików, Poland). Strains were cultivated on Potato Dextrose Agar—PDA (Merck, Darmstadt, Germany) for moulds, and on Tryptic Soy Agar—TSA (Merck, Darmstadt, Germany) for bacteria, and stored at 4 °C.

The spore suspension of fungal strains was prepared by washing out spores from the surface of the pure culture on the PDA medium into 0.85% NaCl with Tween (0.02%) and adjusted to 10^6^ CFU/mL. Bacterial suspension was prepared from the pure culture on the TSA medium and adjusted to 10^6^ CFU/mL.

### 3.3. In Vitro Antimicrobial Assay

The antimicrobial activity of water and water-glycol plant extracts was determined by the agar-well diffusion method. Freshly prepared fungal and bacterial suspensions of 100 µL were inoculated onto PDA (for moulds) or TSA (for bacteria) plates. Then, wells 10 mm in diameter were cut by a sterile cork bore and 250 µL of extracts were introduced into the wells. Water or a water-glycol mixture was used as a control. The plates were incubated for 2–5 days (depending on the pathogen) at 25 °C. The diameters of the phytopathogen growth inhibition zones were measured, excluding the well diameters. The results were expressed as the average of three independent repetitions for each pathogen–extract combination with a standard deviation value.

The antimicrobial activity of subcritical carbon dioxide extracts was determined by the agar-disc diffusion method. Freshly prepared fungal and bacterial suspensions of 100 µL were inoculated onto PDA (for moulds) or TSA (for bacteria) plates. Then, sterile discs 6 mm in diameter were placed on the plates, and 5 µL of the extracts were applied on the discs. The plates were incubated for 2–5 days (depending on the pathogen) at 25 °C. The diameters of phytopathogen growth inhibition zones were measured, excluding the disc diameters. The results were expressed as the average of three independent repetitions for each pathogen–extract combination with a standard deviation value.

Antimicrobial activity was assessed based on Ponce et al. [28]; the diameter of the growth inhibition zone <8 was considered as an inactive substance, 9–15 mm as an active substance, and > 15 mm as a very active substance.

### 3.4. Minimal Inhibitory Concentration (MIC) and Minimal Bactericidal/Fungicidal Concentration (MBC/MFC) Determination

MIC values for garlic and clove water extracts were examined using the macro-broth dilution method. One mL of each extract was serially diluted in 1 mL of malt extract broth medium—MEB (Merck, Darmstadt, Germany) for moulds or tryptic soy broth—TSB (Merck, Darmstadt, Germany) for bacteria to reduce concentration from 50 to 0.098 mg/mL. Then, 1 mL of fresh fungal or bacterial suspension was added to test tubes and incubated for 1 to 3 days (depending on the pathogen) at 25 °C. The visible growth of phytopathogens was assessed in order to determine the MIC value. The cultures were inoculated onto malt extract agar—MEA (Merck, Darmstadt, Germany) or a TSA medium in order to determine MBC/MFC values. The cidal effect was noted when no growth on the MEA or TSA plates was observed. For each pathogen–diluted extract combination, three repetitions were performed.

MIC values for caraway and thyme subcritical carbon dioxide extracts were assessed by the agar-disc diffusion method. Extracts were serially diluted in 96% ethanol (POCH, Gliwice, Poland) in order to reduce concentration from 91.7 to 0.09 mg/mL. Freshly prepared fungal and bacterial suspensions of 100 µL were inoculated onto PDA (for moulds) or TSA (for bacteria) plates. Then, sterile discs 6 mm in diameter were placed on the plates and 5 µL of the diluted extracts was applied on each disc. Ninety-six percent (1:2 diluted, *v*/*v*) ethanol was used as a control. Plates were incubated for 2–5 days (depending on the pathogen) at 25 °C. Pathogen growth around the discs was assessed and MIC values were determined. The MIC value was noted for the lowest extract concentration for which a growth inhibition zone was still observed. For each pathogen–diluted extract combination, 3 repetitions were performed.

### 3.5. Gas Chromatography with Flame Ionisation Detector and Mass Spectrometry of Selected Extracts

Analyses were conducted in a Thermo Ultra GC Trace equipped with a flame ionisation detector and a Thermo DSQ II mass spectrometer (split flow). An Rxi^®^—1 ms (60 m × 0.25 mm × 0.25 µm film thickness) column from RESTEK was used. The injector temperature was 280 °C, and the detector temperature (FID) was 300 °C. The MS temperatures were as follows: transfer line, 280 °C and ion source, 220 °C. The scan range operated between 20 U and 250 U (from 2.50 min to 4.90 min) and between 13 U and 250 U (above 4.90 min). The oven was programmed at 50 °C, held for 3 min; then, a 4 °C/min increase ramped the temperature to 310 °C, where it remained for 10 min. The injection quantity was 1 µL with 15 mL/min split for water extract samples, and 0.5 µL with 100 mL/min split for CO_2_ extract samples. The carrier gas (He) flow was kept constant at 300 kPa. Based on electron spectra from the NIST 2011 library and the Kovats index, the studied analyte was identified. The volatile compounds were identified.

### 3.6. In Situ Antimicrobial Assay on Seed Potatoes

Healthy seed potatoes cv. Impresja obtained from Zamarte Potato Breeding (Zamarte, Poland) were rinsed in sterile distilled water and left to dry in air for 1 h. Three 5 mm in diameter and 5 mm deep cuts were made on each potato using a sterile cork bore. Then, the potatoes were immersed in the extracts and left to dry for 2 h. After that, 20 µL of each fungal or bacterial suspension was applied to the cuts. Control potatoes were immersed in sterile 0.85% NaCl. After 2 weeks of incubation at 25 °C, the percentage of phytopathogen infestation was measured in comparison to the controls according to Equation (1):

(1) $$Reduction\;\left(\%\right)=\frac{C-T}{C}\times \;100$$

where *C* is the % of phytopathogen infestation on control potatoes and *T* is the % of phytopathogen infestation on potatoes treated with extracts. The percentage of the infested area was measured after cutting the seed potatoes in half. The experiments were conducted in triplicate.

### 3.7. Statistical Analysis

Statistical analysis was carried out with Statistica 13.1 (Statsoft, Tulsa, OK, USA). The antimicrobial activity (phytopathogen growth inhibition zones) of all tested plants among three different types of extracts was compared using a one-way analysis of variance (ANOVA) at a significance level of 0.05. When a statistical difference was detected (*p* < 0.05), the means were compared using Tukey’s post hoc procedure at a significance level of 0.05.

## 4. Conclusions

Our research showed that water extracts obtained from sage, garlic, clove, onion, turmeric, and bistort contain compounds that are active against at least one species of potato phytopathogens. The greatest potential for use in the development of a protection regimen for seed potatoes during storage was demonstrated by garlic water extract. It exhibited its broad spectrum of antimicrobial activity against phytopathogens in vitro and its highest degree of phytopathogen growth reduction in situ. A significant advantage of using water extracts is also the low cost of their production. Due to differences in the antimicrobial activity of garlic water extract against potato phytopathogens in vitro and in situ, further research should focus on modifying the methods of applying the extract in order to obtain a wider spectrum of action.

## Figures and Tables

**Table 1:** MIC and MBC/MFC values of selected water and subcritical carbon dioxide extracts (SCDE) against potato phytopathogens.

Phytopathogens	Water Extracts				SCDE	
	*Allium sativum*		*Caryophyllus aromaticus*		*Thymus vulgaris*	*Carum carvi*
	MIC *^a^*	MBC/MFC	MIC *^a^*	MBC/MFC	MIC *^b^*	
	[mg/mL]				[mg/mL EtOH]	
*Fusarium oxysporum*	12.5	25.0	6.3	12.5	5.7	22.5
*Fusarium sambucinum*	12.5	25.0	3.1	12.5	5.7	nd
*Alternaria alternata*	25.0	25.0	6.3	nd	11.5	90.0
*Alternaria solani*	6.3	25.0	3.1	6.3	5.7	nd
*Alternaria tenuissima*	25.0	nd	6.3	12.5	11.5	90.0
*Colletotrichum coccodes*	12.5	25.0	6.3	12.5	2.9	45.0
*Rhizoctonia solani*	12.5	12.5	3.1	6.3	5.7	45.0
*Phoma exigua*	6.3	12.5	3.1	6.3	11.5	90.0
*Pectobacterium carotovorum*	25.0	25.0	6.3	25.0	5.7	90.0
*Streptomyces scabiei*	12.5	25.0	3.1	12.5	2.9	90.0

^a^—determined by the macro-broth dilution method; ^b^—determined by the agar-disc diffusion method; MIC—Minimal Inhibitory Concentration; MBC/MFC—Minimal Bactericidal Concentration/Minimal Fungicidal Concentration; nd—not detected.

**Table 2:** Volatile compounds of *Allium sativum* L. water extract.

Component	KI	Composition [%]
Acetaldehyde		5.17
Methyl mercaptan		0.36
Propionaldehyde	512	1.60
2-Methylpropanal	557	0.88
Butane-2,3-dione	584	0.32
Allyl mercaptan	599	3.52
3-Methylbutanal	650	4.27
Acetic acid	655	1.93
1-Hydroxypropan-2-one	676	5.46
1-(Methylthio)prop-1-ene	688	0.22
Methyl prop-2-enoate	742	0.21
3-Hydroxypropanal	752	0.25
Mercaptoacetone	765	1.68
Furan-2-carboxaldehyde	804	7.09
Furan-2-methanol	840	0.25
Diallyl sulphide	841	0.30
Dihydrofuran-2(3H)-one	866	0.56
Allyl propyl sulphide	888	0.38
Methyl prop-2-enyl disulphide	894	0.44
3-Methylfuran-2(5H)-one	897	1.19
1-(Ethylthio)-2-methylprop-1-ene	918	0.17
5-Methylfuran-2-carboxaldehyde	929	0.81
Dimethyl trisulphide	947	0.95
2,4-Dihydroxy-2,5-dimethylfuran-3(2H)-one	957	0.13
3-Methylcyclopentane-1,2-dione	995	0.28
Benzeneacetaldehyde	1006	0.43
Butyl angelate	1034	0.16
2,5-Dimethyl-4-hydroxyfuran-3(2H)-one	1040	2.25
2,3-Dihydro-5-hydroxy-6-methyl-4H-pyran-4-one	1049	0.51
Diallyl disulphide	1056	1.58
Linalool	1085	0.50
Allyl methyl trisulphide	1111	0.41
Pyranone	1118	3.73
l-Pinocarveol	1123	0.18
Isoamyl angelate	1136	0.10
Pinocarvone	1138	0.10
3-Vinyl-1,2-dithiacyclohex-4-ene	1157	0.44
3-Vinyl-1,2-dithiacyclohex-5-ene	1178	0.22
5-Hydroxymethylfurfural	1196	33.24
(+)-Carvone	1216	0.19
Di-2-propenyl trisulphide	1273	0.18
Palmitic acid	1772	0.32
Total unidentified compounds	–	16.99

KI—Kovats index.

**Table 3:** Volatile compounds of *Caryophyllus aromaticus* L. water extract.

Component	KI	Composition [%]
Acetaldehyde		0.38
Ethyl acetate	618	0.25
Acetic acid	650	0.22
2-Methylbutanal	659	0.15
1-Hydroxypropan-2-one	686	0.11
Propylene glycol	727	0.24
Hex-3-en-1-ol	841	0.65
Hexan-1-ol	855	0.16
Octan-3-ol	981	0.13
Benzeneacetaldehyde	1008	0.15
Eucalyptol	1019	0.08
Butyl angelate	1035	0.18
p-Cresol	1055	0.72
Linalool	1085	0.58
α-Thujone	1097	0.09
Pyranone	1115	0.18
Camphor	1120	0.22
l-Pinocarveol	1123	0.63
Camphene hydrate	1132	0.09
Isoamyl tiglate	1137	0.11
Pinocarvone	1138	0.27
Borneol	1150	0.33
Terpinen-4-ol	1162	0.34
α-Terpineol	1173	0.42
Carvone	1216	0.61
3-Methylpentylangelate	1236	0.05
Eugenol	1334	82.39
Caryophyllene	1419	2.49
α-Humulene	1452	0.23
Eugenol acetate	1486	4.56
Selina-6-en-4-ol	1641	0.10
Hexadecanoic acid	1941	0.41
Stearic acid	2141	0.11
Total unidentified compounds	–	2.46

KI—Kovats index.

**Table 4:** Volatile compounds of *Thymus vulgaris* L. subcritical carbon dioxide extract.

Component	KI	Composition [%]
Methyl 2-methylbutanoate	757	0.03
α-Thujene	920	0.14
α-Pinene	927	0.18
Camphene	940	0.11
Oct-1-en-3-ol	962	0.78
β-Pinene	967	0.08
Myrcene	980	0.55
α-Phellandrene	994	0.05
Car-3-ene	1002	0.02
iso-Terpinene	1007	0.52
*p*-Cymene	1012	8.53
Eucalyptol	1018	0.51
d-Limonene	1019	0.19
γ-Terpinen	1049	4.10
4-Thujanol	1053	0.87
*p*-Cymenene	1073	0.02
Terpinolene	1078	0.03
4-Thujanol	1082	0.14
Linalool	1086	1.93
Camphor	1119	0.09
endo-Borneol	1149	0.50
Terpinen-4-ol	1162	0.20
Menthol	1170	0.04
α-Terpineol	1183	0.14
Thymol methyl ether	1215	0.43
2-isopropyl-4-methyl-anisole	1226	0.31
Thymoquinone	1228	0.04
(+)-Carvone	1233	0.17
Geraniol	1253	0.07
Thymol	1283	48.54
Carvacrol	1288	3.16
Copaene	1377	0.05
(-)-β-Bourbonene	1385	0.04
Thymohydroquinone	1420	4.57
Caryophyllene	1421	1.59
Geranyl propanoate	1453	0.22
γ-Muurolene	1472	0.16
β-Bisabolene	1502	0.08
γ-Cadin-2-ene	1509	0.25
cis-Calamenene	1512	0.06
δ-Cadinene	1517	0.30
Caryophyllene oxide	1575	0.59
Humulene epoxide II	1598	0.05
10-epi-γ-eudesmol	1612	0.06
τ-Cadinol	1629	0.21
(-)-Loliolide	1707	0.02
Oplopanone	1712	0.02
Myristic acid	1742	0.05
Neophytadiene	1837	0.19
Hexadecan-1-ol	1864	0.09
3,7,11,15-Tetramethylhexadec-2-en-1-ol	1879	0.06
Hexadecanoic acid	1960	5.13
Phytol	2102	0.37
Oleic acid	2133	6.34
Stearic acid	2154	0.50
Arachidic acid	2345	0.06
Glyceryl Linolenate	2438	0.07
Glyceryl Monooleate	2444	0.19
Squalene	2805	0.21
Nonacosane	2881	0.33
Triacontane	3068	0.17
Vitamin E	3084	0.43
γ-Sitosterol	3324	0.33
Total unidentified compounds	–	4.74

KI—Kovats index.

**Table 5:** Volatile compounds of *Carum carvi* L. subcritical carbon dioxide extract.

Component	KI	Composition [%]
(+)-Sabinene	963	0.03
Myrcene	980	0.25
d-Limonene	1025	37.17
Car-3-ene	1037	0.05
γ-Terpinen	1048	0.04
Linalool	1084	0.05
Trans-p-mentha-2,8-dienol	1103	0.08
Cis-limonene oxide	1116	0.14
Trans-Limonene oxide	1120	0.09
Menthol	1158	0.05
Trans-dihydrocarvone	1178	0.08
Trans-carveol	1208	0.11
(+)-Carvone	1227	52.14
(E)-Citral	1245	0.02
Perillaldehyde	1248	0.24
Methyl geraniate	1302	0.02
Caryophyllene	1419	0.25
α-Humulene	1452	0.02
Germacrene	1478	0.02
Caryophyllene oxide	1573	0.04
Neophytadiene	1837	0.09
Hexadecanoic acid	1947	1.19
Phytol	2100	0.04
Linoleic acid	2117	1.82
Oleic acid	2130	3.34
Octadecanoic acid	2147	0.22
Arachidic acid	2344	0.03
2-Glyceryl linoleate	2387	0.06
2,3-Dihydroxypropyl elaidate	2394	0.12
Glyceryl linolenate	2438	0.23
Glyceryl monooleate	2445	0.57
Squalene	2805	0.24
Nonacosane	2881	0.34
Total unidentified compounds	–	0.81

KI—Kovats index.

**Table 6:** Reduction of potato seed cv. Impresja infestation treated with selected plant extracts against potato phytopathogens.

Phytopathogens	Reduction of Potato Infestation [%]			
	Water Extracts		SCDE	
	*Allium sativum* L.	*Caryophyllus aromaticus* L.	*Thymus vulgaris* L.	*Carum carvi* L.
*Fusarium oxysporum*	100.0	88.9 ± 1.97	33.3 ± 1.88	55.6 ± 0.63
*Fusarium sambucinum*	100.0	0.0	nd	nd
*Alternaria alternata*	0.0	0.0	nd	nd
*Alternaria solani*	100.0	+25.0 ± 0.0 *	nd	nd
*Alternaria tenuissima*	0.0	100.0	nd	nd
*Colletotrichum coccodes*	100.0	+10.0 ± 2.89 *	55.6 ± 3.1	83.3 ± 1.89
*Rhizoctonia solani*	100.0	+93.8 ± 1.64 *	nd	nd
*Phoma exigua*	100.0	+95.0 ± 0.0 *	nd	nd
*Pectobacterium carotovorum*	0.0	0.0	0.0	0.0
*Streptomyces scabiei*	100.0	+75.0 ± 2.5 *	nd	nd

nd—not determined; *—potato infestation increase.

**Table 7:** Plant material used for the preparation of extracts.

Latin Name	Common Name	Part of the Plant
*Achillea millefolium* L.	Common yarrow	Leaves, stems
*Mentha piperita* L.	Peppermint	Leaves, stems
*Salvia officinalis* L.	Sage	Leaves, stems
*Equisetum arvense* L.	Common horsetail	Leaves, stems
*Urtica dioica* L.	Nettle	Leaves, stems
*Taraxacum officinale* (L.) Weber ex F.H. Wigg.	Dandelion	Leaves, stems
*Elymus repens* (L.) Gould	Couch grass	Leaves, stems
*Hypericum perforatum* L.	Perforate St. John’s wort	Root
*Rosmarinus officinalis* Spenn.	Rosemary	Leaves, stems
*Humulus lupulus* L.	Common hop	Inflorescences
*Satureja hortensis* L.	Summer savoury	Leaves, stems
*Carum carvi* L.	Caraway	Seeds
*Nigella sativa* L.	Blackseed	Seeds
*Thymus vulgaris* L.	Thyme	Seeds
*Lavandula angustifolia* Mill.	Lavender	Flower buds
*Armoracia rusticana* G. Gaertn., B. Mey. & Scherb.	Horseradish	Root
*Allium sativum* L.	Garlic	Bulb
*Syzygium aromaticum* (L.) Merr. & Perry	Clove	Flower buds
*Allium cepa* L.	Onion	Bulb
*Curcuma longa* L.	Turmeric	Root
*Polygonum bistorta* L.	Bistort	Leaves, stems
*Polygonum aviculare*	Common knotgrass	Leaves, stems

## Supplementary Materials

The following supporting information can be downloaded online, Table S1: Activity of plant water extracts against potato seed phytopathogens measured as growth inhibition zones in the agar-well diffusion method; Table S2: Activity of plant glycol-water extracts against potato seed phytopathogens measured as growth inhibition zones in the agar-well diffusion method; Table S3: Activity of plant subcritical carbon dioxide extracts against potato seed phytopathogens measured as growth inhibition zones in the agar-disc diffusion method.
