# Supplementary material for: Antimicrobial Activities of Plant Extracts against Solanum tuberosum L. Phytopathogens
Source: Molecules. 2022 Feb 27;27(5):1579. doi: 10.3390/molecules27051579 (PMC8911893; doi:10.3390/molecules27051579)
Supplement: Supplementary file 1 [file molecules-27-01579-s001.zip › molecules-1596218-supplementary.pdf]

Table S1. Activity of plant water extracts against potato seed phytopathogens measured as growth inhibition zones in the agar-well diffusion method. M – mean; SD – standard deviation; a-b —the same letter is not significantly different (ANOVA. α = 0.05; Tukey's test. α= 0.05).

**Plant species**	**Phytopathogen growth inhibition zone M+SD [mm]**									
	Fusarium oxysporum	***Fusarium sambucinum***	***Alternaria alternata***	***Alternaria solani***	***Alternaria tenuissima***	***Colletotrichum coccodes***	***Rhizoctonia solani***	***Phoma exigua***	***Pectobacterium carotovorum***	***Streptomyces scabiei***
Achillea millefolium[a]	0.0±0.0	0.0±0.0	0.0±0.0	0.0±0.0	0.0±0.0	0.0±0.0	0.0±0.0	0.0±0.0	0.0±0.0	0.0±0.0
Mentha piperita[a]	0.0±0.0	0.0±0.0	0.0±0.0	0.0±0.0	0.0±0.0	0.0±0.0	0.0±0.0	0.0±0.0	0.0±0.0	0.0±0.0
Salvia officinalis[a]	11.0±1.0	0.0±0.0	0.0±0.0	0.0±0.0	0.0±0.0	0.0±0.0	0.0±0.0	0.0±0.0	0.0±0.0	0.0±0.0
Equisetum arvense[a]	0.0±0.0	0.0±0.0	0.0±0.0	0.0±0.0	0.0±0.0	0.0±0.0	0.0±0.0	0.0±0.0	0.0±0.0	0.0±0.0
Urtica dioica[a]	0.0±0.0	0.0±0.0	0.0±0.0	0.0±0.0	0.0±0.0	0.0±0.0	0.0±0.0	0.0±0.0	0.0±0.0	0.0±0.0
Taraxacum officinale[a]	0.0±0.0	0.0±0.0	0.0±0.0	0.0±0.0	0.0±0.0	0.0±0.0	0.0±0.0	0.0±0.0	0.0±0.0	0.0±0.0
Elymus repens[a]	0.0±0.0	0.0±0.0	0.0±0.0	0.0±0.0	0.0±0.0	0.0±0.0	0.0±0.0	0.0±0.0	0.0±0.0	0.0±0.0
Hypericum perforatum[a]	0.0±0.0	0.0±0.0	0.0±0.0	0.0±0.0	0.0±0.0	0.0±0.0	0.0±0.0	0.0±0.0	0.0±0.0	0.0±0.0
Rosmarinus officinalis[a]	0.0±0.0	0.0±0.0	0.0±0.0	0.0±0.0	0.0±0.0	0.0±0.0	0.0±0.0	0.0±0.0	0.0±0.0	0.0±0.0
Humulus lupulus[a]	0.0±0.0	0.0±0.0	0.0±0.0	0.0±0.0	0.0±0.0	0.0±0.0	0.0±0.0	0.0±0.0	0.0±0.0	0.0±0.0
Satureja hortensis[a]	0.0±0.0	0.0±0.0	0.0±0.0	0.0±0.0	0.0±0.0	0.0±0.0	0.0±0.0	0.0±0.0	0.0±0.0	0.0±0.0
Carum carvi[a]	0.0±0.0	0.0±0.0	0.0±0.0	0.0±0.0	0.0±0.0	0.0±0.0	0.0±0.0	0.0±0.0	0.0±0.0	0.0±0.0
Nigella sativa[a]	0.0±0.0	0.0±0.0	4.0±1.0	0.0±0.0	1.0±0.0	5.3±0.6	1.7±0.6	0.0±0.0	4.7±0.6	0.0±0.0
Thymus vulgaris[a]	1.7±0.6	0.0±0.0	0.0±0.0	1.3±0.6	0.0±0.0	0.0±0.0	0.0±0.0	0.0±0.0	0.0±0.0	0.0±0.0
Lavandula angustifolia[a]	0.0±0.0	0.0±0.0	0.0±0.0	0.0±0.0	0.0±0.0	0.0±0.0	0.0±0.0	0.0±0.0	0.0±0.0	0.0±0.0
Armoracia rusticana[a]	0.0±0.0	0.0±0.0	0.0±0.0	0.0±0.0	0.0±0.0	0.0±0.0	0.0±0.0	0.0±0.0	0.0±0.0	0.0±0.0
Allium sativum[b]	39.7±0.6	19.7±0.6	24.3±0.6	49.7±0.6	31.0±1.0	41.3±0.6	56.0±1.0	43.3±	25.0±1.0	27.7±0.6
Caryophyllus aromaticus[b]	25.7±0.6	11.0±	28.7±1.2	49.0±1.0	25.7±0.6	25.3±0.6	57.7±0.6	39.7±0.6	20.7±0.6	41.7±0.6
Allium cepa[a]	0.0±0.0	0.0±0.0	0.0±0.0	0.0±0.0	0.0±0.0	0.0±0.0	0.0±0.0	20.7±0.6	0.0±0.0	0.0±0.0
Curcuma longa[a]	0.0±0.0	0.0±0.0	0.0±0.0	0.0±0.0	0.0±0.0	0.0±0.0	0.0±0.0	0.0±0.0	0.0±0.0	29.7±0.6
Polygonum bistorta[a]	0.0±0.0	0.0±0.0	0.0±0.0	0.0±0.0	0.0±0.0	0.0±0.0	0.0±0.0	0.0±0.0	0.0±0.0	24.7±0.6
Polygonum aviculare[a]	0.0±0.0	0.0±0.0	0.0±0.0	0.0±0.0	0.0±0.0	0.0±0.0	0.0±0.0	0.0±0.0	0.0±0.0	0.0±0.0

Table S2. Activity of plant glycol-water extracts against potato seed phytopathogens measured as growth inhibition zones in the agar-well diffusion method. M – mean; SD – standard deviation; a-e —the same letter is not significantly different (ANOVA. α = 0.05; Tukey's test. α= 0.05).

**Plant species**	**Phytopathogen growth inhibition zone M+SD [mm]**									
	Fusarium oxysporum	***Fusarium sambucinum***	***Alternaria alternata***	***Alternaria solani***	***Alternaria tenuissima***	***Colletotrichum coccodes***	***Rhizoctonia solani***	***Phoma exigua***	***Pectobacterium carotovorum***	***Streptomyces scabiei***
Achillea millefolium^{ab}	0.0±0.0	0.0±0.0	0.0±0.0	0.0±0.0	0.0±0.0	0.0±0.0	0.0±0.0	12.01.0	3.0±1.0	11.7±1.2
Mentha piperita^{ab}	0.0±0.0	0.0±0.0	0.0±0.0	0.0±0.0	0.0±0.0	0.0±0.0	19.3±0.6	0.0±0.0	5.7±0.6	0.0±0.0
Salvia officinalis^{abc}	3.3±0.6	0.0±0.0	7.3±0.6	6.0±1.0	6.7±0.6	1.2±0.6	21.7±4.2	6.0±1.0	7.0±1.0	24.0±1.0
Equisetum arvense^{ab}	0.0±0.0	0.0±0.0	3.7±0.6	0.0±0.0	0.0±0.0	0.0±0.0	12.0±1.0	0.0±0.0	0.0±0.0	16.3±1.2
Urtica dioica^{a}	0.0±0.0	0.0±0.0	0.0±0.0	0.0±0.0	0.0±0.0	0.0±0.0	6.3±1.2	0.0±0.0	0.0±0.0	0.0±0.0
Taraxacum officinale^{a}	0.0±0.0	0.0±0.0	0.0±0.0	0.0±0.0	0.0±0.0	0.0±0.0	0.0±0.0	0.0±0.0	5.0±0.0	13.7±
Elymus repens^{a}	0.0±0.0	0.0±0.0	0.0±0.0	0.0±0.0	0.0±0.0	0.0±0.0	3.7±1.2	3.7±1.2	0.0±0.0	9.7±0.6
Hypericum perforatum^{ab}	0.0±0.0	0.0±0.0	0.0±0.0	0.0±0.0	0.0±0.0	6.7±0.6	0.0±0.0	6.0±1.0	5.0±0.0	15.0±0.0
Rosmarinus officinalis^{ab}	0.0±0.0	0.0±0.0	0.0±0.0	0.0±0.0	0.0±0.0	0.0±0.0	18.0±1.7	1.7±0.6	7.0±0.0	22.0±1.7
Humulus lupulus^{abc}	1.3±0.6	2.7±0.6	4.3±1.2	0.0±0.0	4.7±0.6	0.0±0.0	1.3±0.6	9.0±1.0	5.0±0.0	25.0±2.0
Satureja hortensis^{ab}	0.0±0.0	0.0±0.0	0.0±0.0	0.0±0.0	0.0±0.0	5.0±0.0	13.7±1.2	2.3±0.6	9.0±0.0	20.7±0.6
Carum carvi^{a}	0.0±0.0	0.0±0.0	1.0±0.0	0.0±0.0	0.0±0.0	0.0±0.0	13.0±2.0	0.0±0.0	6.0±1.0	7.3±0.6
Nigella sativa^{ab}	0.0±0.0	0.0±0.0	10.0±	0.0±0.0	0.0±0.0	6.7±0.6	7.7±2.5	14.7±0.6	8.7±1.2	0.0±0.0
Thymus vulgaris^{ab}	0.0±0.0	5.0±1.0	7.3±0.6	3.0±1.7	0.0±0.0	9.7±0.6	12.7±2.1	2.3±0.6	3.7±1.2	0.0±0.0
Lavandula angustifolia^{ab}	3.7±0.6	4.3±1.2	0.0±0.0	0.0±0.0	0.0±0.0	9.7±0.6	0.0±0.0	0.0±0.0	5.3±0.6	0.0±0.0
Armoracia rusticana^{ab}	0.0±0.0	0.0±0.0	0.0±0.0	0.0±0.0	0.0±0.0	2.7±0.6	0.0±0.0	0.0±0.0	9.1±0.6	12.7±0.6
Allium sativum^{d}	29.7±0.6	0±0.0	29.7±0.6	51.0±1.0	26.7±0.6	34.7±0.6	33.3±1.5	51.0±1.0	25.7±0.6	35.7±0.6
Caryophyllus aromaticus^{e}	49.0±1.0	41.0±1.0	34.7±0.6	59.0±1.0	40.3±0.6	50.3±0.6	52.7±2.5	55.0±1.0	26.3±0.6	49.3±0.6
Allium cepa^{ab}	0.0±0.0	0.0±0.0	0.0±0.0	0.0±0.0	0.0±0.0	0.0±0.0	0.0±0.0	0.0±0.0	21.3±0.6	0.0±0.0
Curcuma longa^{c}	0.0±0.0	0.0±0.0	0.0±0.0	18.3±0.6	0.0±0.0	18.7±0.6	24.3±0.6	21.3±0.6	0.0±0.0	28.3±0.6
Polygonum bistorta^{bc}	0.0±0.0	0.0±0.0	22.3±0.6	0.0±0.0	0.0±0.0	16.7±0.6	0.0±0.0	25.0±1.0	22.0±1.0	0.0±0.0
Polygonum aviculare^{abc}	0.0±0.0	0.0±0.0	17.7±0.6	0.0±0.0	0.0±0.0	0.0±0.0	0.0±0.0	0.0±0.0	21.7±0.6	23.7±1.5

Table S3. Activity of plant subcritical carbon dioxide extracts against potato seed phytopathogens measured as growth inhibition zones in the agar-disc diffusion method. M – mean; SD – standard deviation; a-b —the same letter is not significantly different (ANOVA. $\alpha = 0.05$; Tukey's test. $\alpha = 0.05$).

**Plant species**	**Phytopathogen growth inhibition zone M+SD [mm]**									
	Fusarium oxysporum	***Fusarium sambucinum***	***Alternaria alternata***	***Alternaria solani***	***Alternaria tenuissima***	***Colletotrichum coccodes***	***Rhizoctonia solani***	***Phoma exigua***	***Pectobacterium carotovorum***	***Streptomyces scabiei***
Carum carvi[a]	5.0±0	0.0±0.0	15.3±0.6	0.0±0.0	7.3±	10.3±0.6	7.3±0.6	12.7±0.6	4.0±0.0	10.0±1.0
Thymus vulgaris[b]	25.3±0.6	45.0±1.0	15.3±1.2	35.3±0.6	13.3±0.6	45.3±1.2	45.3±1.5	48.7±1.2	10.0±1.0	21.3±1.2
Nigella sativa[a]	9.3±1.5	11.3±0.6	15.0±1.0	2.7±0.6	0.0±0.0	16.0±1.0	0.0±0.0	0.0±0.0	0.0±0.0	19.3±1.2
